# A Case of Aplastic Anemia Complicated With Cerebral Infarction

**DOI:** 10.7759/cureus.39274

**Published:** 2023-05-20

**Authors:** Sanchit S Chouksey, Abhishek Pathak, Vijay Nath Mishra, Nikhil A Kumar, Rohit Daga

**Affiliations:** 1 Department of Neurology, Institute of Medical Sciences, Banaras Hindu University, Varanasi, IND; 2 Department of General Medicine, Institute of Medical Sciences, Banaras Hindu University, Varanasi, IND

**Keywords:** young onset stroke, cortical stroke, cerebral infraction, aplastic, anemia

## Abstract

Aplastic anemia is usually associated with hemorrhagic stroke. Here, we report a case of ischemic stroke secondary to aplastic anemia in a 28-year-old male who presented with sudden-onset right hemiplegia and aphasia when he was not on any immunosuppression for five months. His laboratory findings showed pancytopenia, and his peripheral blood smear showed no atypical cells. Magnetic resonance imaging of the brain with magnetic resonance angiography (MRA) of the neck and brain vessels revealed an infarct in the left cerebral hemisphere in the middle cerebral artery territory, and no significant stenosis or aneurysm was observed on MRA. The patient was managed conservatively and discharged in stable condition.

## Introduction

Aplastic anemia (AA) is a rare hemopoietic stem cell disorder that results in pancytopenia and is caused by hypocellular bone marrow. It may be inherited or acquired. The immune-mediated mechanism plays a crucial role in the development of acquired AA. Cells of adaptive immunity mediate the destruction of hemopoietic stem cells which can be triggered by environmental exposure to drugs, viruses, and toxins [1].

AA severity, patient’s age, donor availability, and access to treatment may affect the management of AA. Allogenic bone marrow transplant is the first-line treatment in pediatric and young patients whenever matched donor is available. The treatment of choice for the remaining patients is immunosuppressive therapy (IST) with horse anti-thymocyte globulin (ATG) and cyclosporine A [2].

There are few case reports of AA with intracerebral hemorrhage and cerebral infarction with immunosuppression [3]. Acute myocardial infarction has also been reported in two cases of AA on treatment with anabolic steroids [4]. A case of acquired AA with recurrent cerebral infarctions at the induction of IST has also been described [5]. Here, we report a case of cerebral infarction in AA not related to immunosuppression.

## Case presentation

**Figure 1:**
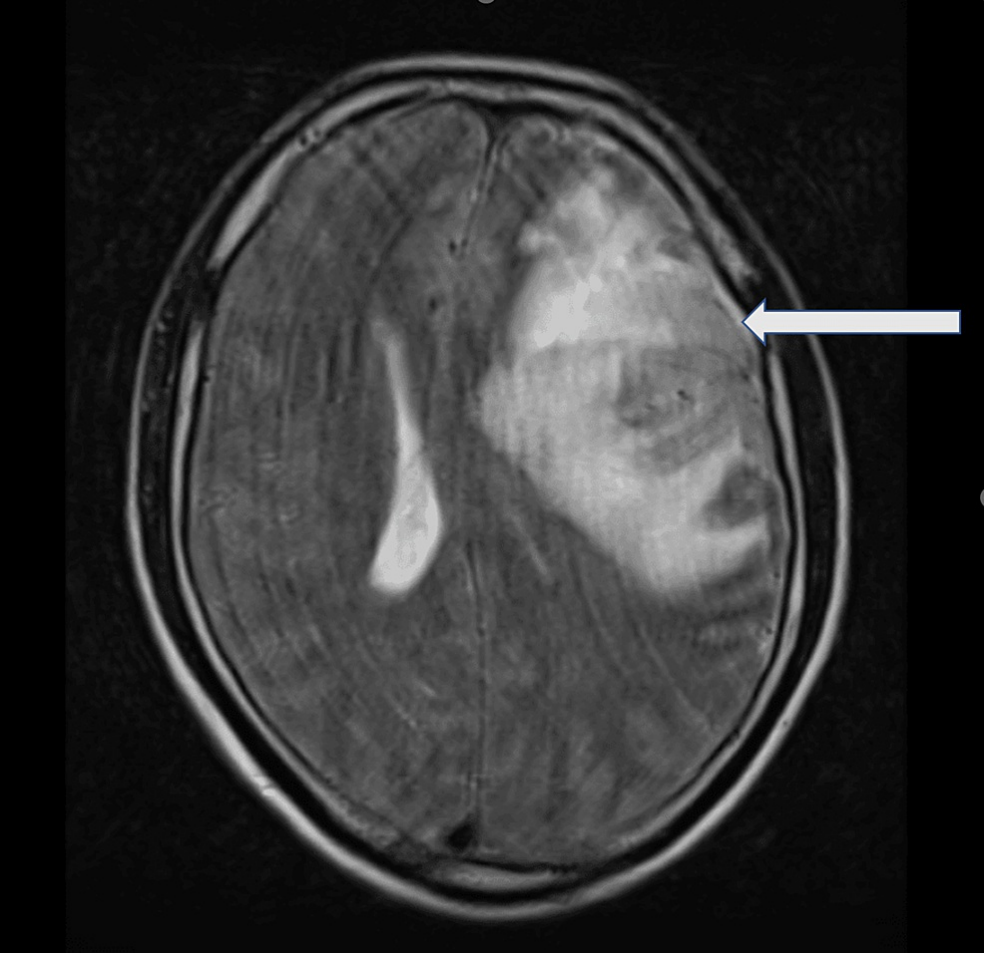
T2-weighted magnetic resonance imaging of the brain. The magnetic resonance imaging shows hyperintensity in the left middle cerebral artery territory suggestive of an acute infarct.

**Figure 2:**
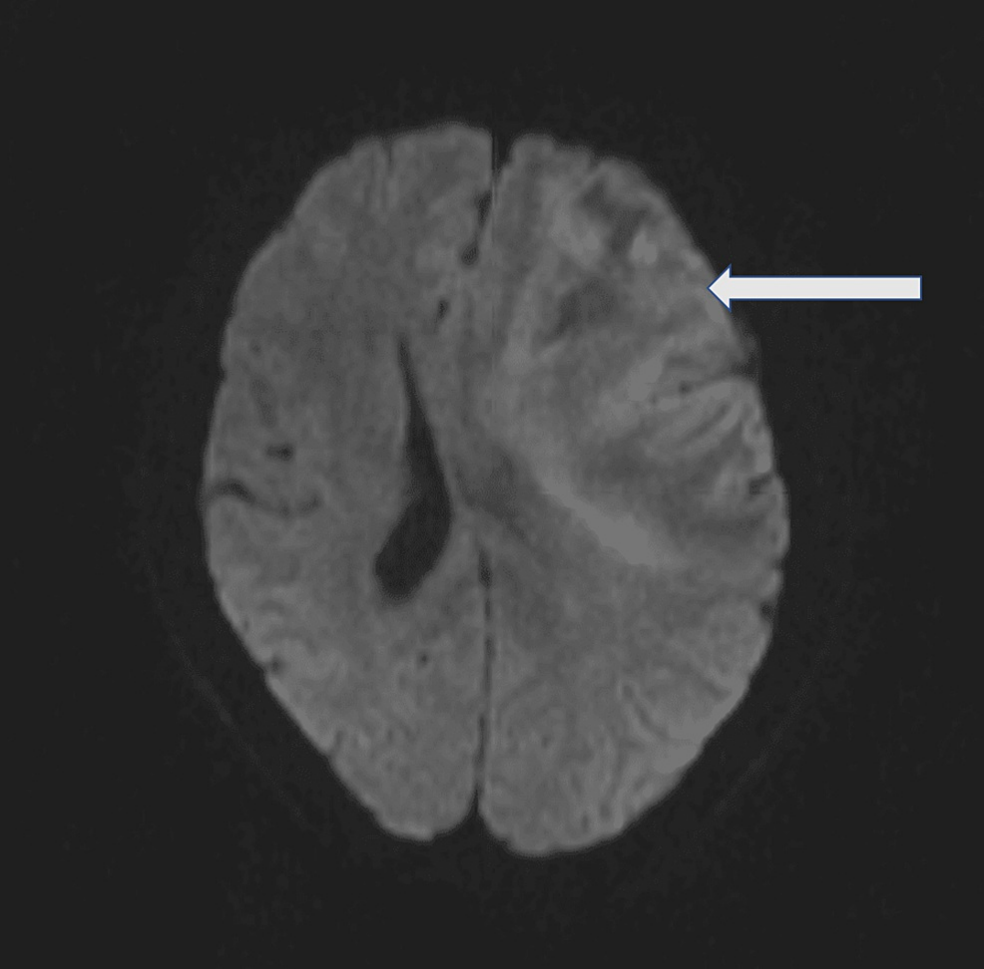
Diffusion-weighted imaging. The magnetic resonance imaging shows diffusion restriction in the left middle cerebral artery territory.

**Figure 3:**
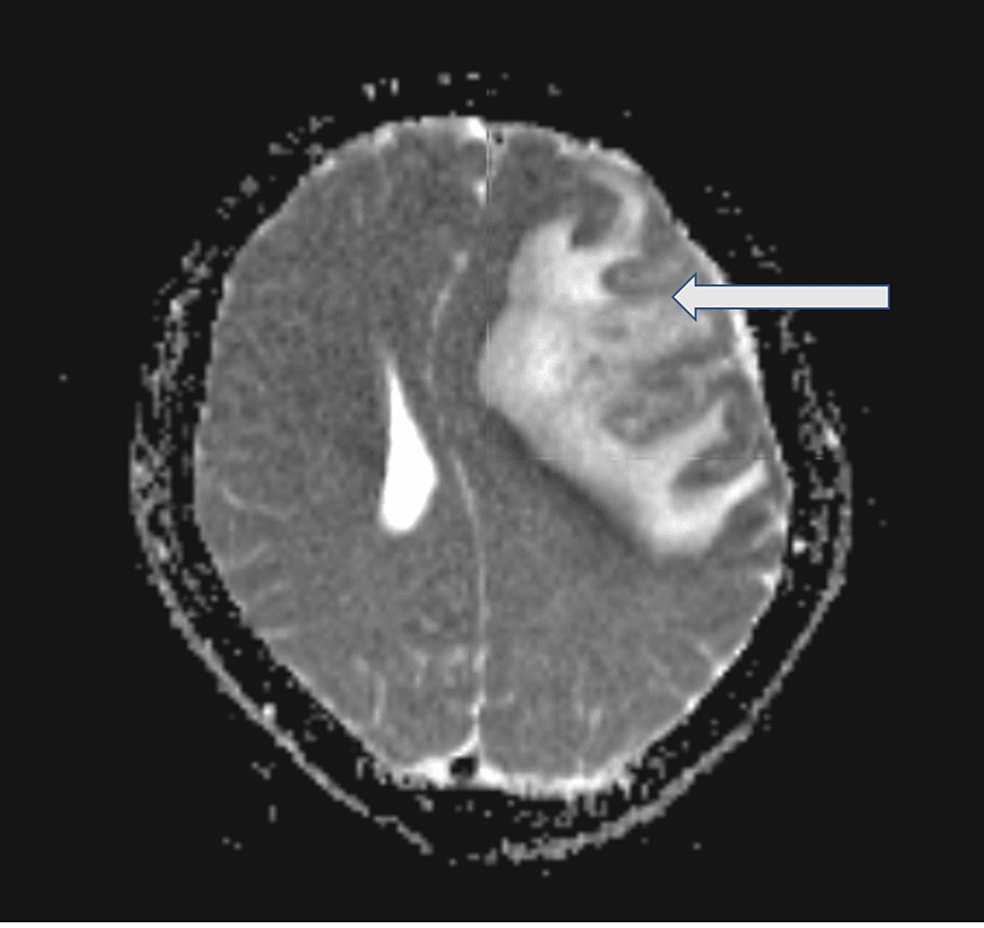
Apparent diffusion coefficient sequence. The magnetic resonance imaging shows an acute left middle cerebral artery infarct.

**Figure 4:**
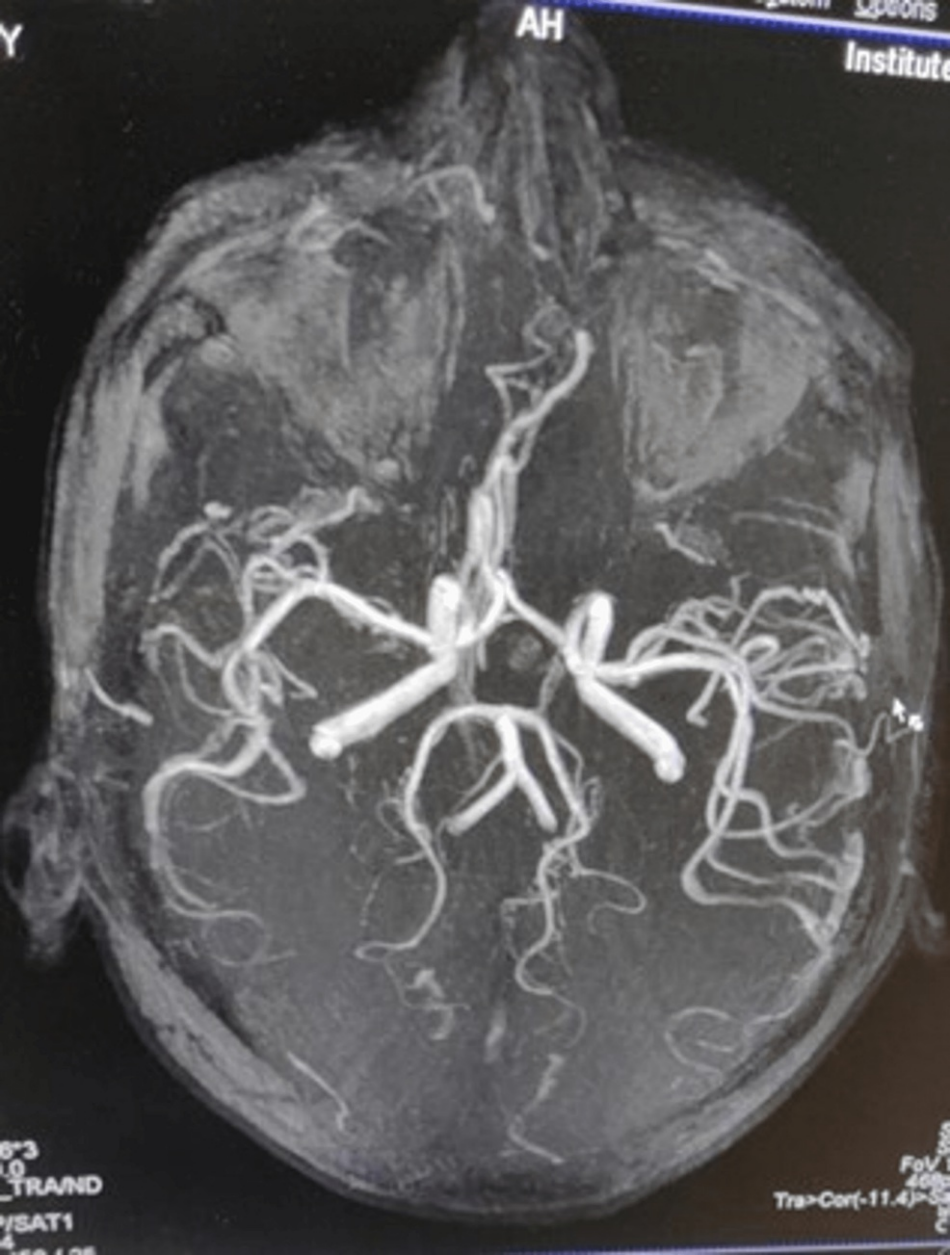
Magnetic resonance angiography of brain vessels. The magnetic resonance angiography shows normal study.

A 28-year-old male who was diagnosed with AA in June 2018 based on a bone marrow biopsy report received ATG therapy in July 2018 as no matched donor was found for a bone marrow transplant. He acquired hepatitis B secondary to transfusion and was adequately treated with tenofovir 300 mg for 11 months. He subsequently became hepatitis B surface antigen negative. The patient was diagnosed with ATG relapse in March 2020 and was maintained on cyclosporine, stanozolol (until February 2022), packed red blood cells, and platelet transfusion. He presented to our hospital with sudden-onset, right-sided hemiplegia and Broca’s aphasia in July 2022 without any prior history of hypertension, diabetes mellitus, and coronary artery disease. His laboratory values at the time of admission were hemoglobin of 6.8 g/dL, total leukocyte count of 2,200 cells/mm^3^, and platelets of 68,000/mm^3^. Non-contrast computed tomography of the head showed a large hypodense lesion in the left cerebral hemisphere in the middle cerebral artery (MCA) territory. It was followed by magnetic resonance imaging of the brain with magnetic resonance angiography (MRA) of the neck and brain vessels which revealed a large infarct in the left cerebral hemisphere in MCA territory and no significant stenosis, obstruction, or aneurysm in the anterior cerebral arteries, MCAs, and posterior cerebral arteries on MRA, as shown in Figures 1-4.

Two-dimensional echocardiography with bubble study was normal ruling out the right to left shunt and the possibility of embolic stroke. The patient was managed conservatively with a short course of mannitol, 75 mg aspirin per day, low-dose statin, and physiotherapy.

The patient improved in due course of hospitalization, his power became 3/5 in the right upper and lower limbs, but his aphasia persisted. The patient was discharged home on statins on day 14 of his illness.

## Discussion

AA is described as bone marrow failure syndrome characterized by pancytopenia and marrow hypoplasia. Due to malfunctioning of all cell lines of blood, patients present with a wide variety of symptoms such as yellowing of eyes, shortness of breath, altered stool color, spleen enlargement, palpitations, and effects on the central nervous system such as dizziness. The ultimate and definitive treatment for AA is a bone marrow transplant [2,6].

IST is used as a temporary measure when arrangements for the transplant are being made or when the option of transplant is not available. Temporary IST is administered with drugs such as ATG, cyclosporine, and tacrolimus [7]. Other important treatment options include blood transfusions [8] and early prevention and treatment of infections such as parvovirus [9].

Multiple studies have shown that after initiating the treatment for AA with IST, patients can present with symptoms of cerebral infarction at the initial stages of therapy [3]. Our presented case describes a patient who developed a cerebral infarction even when he did not use IST for the past five months. Thrombolysis was not done as his platelet count was less than 100,000/µL [10]. The patient was treated with conservative approaches such as head elevation and intravenous mannitol to reduce intracranial pressure [11]. This is the first reported case of this nature in the literature where a patient with AA developed cerebral infarction even when he had abandoned his immunosuppression drugs. Whether the stroke is related to AA is debatable and could be very well by chance.

## Conclusions

In summary, patients with AA are prone to hemorrhagic stroke due to low platelet count. The present case is a rare entity as the patient was off immunosuppression and presented with cerebral infarction; therefore, AA patients should undergo a detailed clinical examination and head imaging if indicated for the early detection of a cerebral infraction. Further studies are required to elucidate the mechanism behind the infarction in AA.
